# A Novel Chinese Herbal and Corresponding Chemical Formula for Cancer Treatment by Targeting Tumor Maintenance, Progression, and Metastasis

**DOI:** 10.3389/fphar.2022.907826

**Published:** 2022-05-26

**Authors:** Ying-Chyi Song, Der-Yen Lee, Pei-Yen Yeh

**Affiliations:** ^1^ Graduate Institute of Integrated Medicine, College of Chinese Medicine, China Medical University, Taichung, Taiwan; ^2^ TCM division, Jin-Mi company, Taipei, Taiwan

**Keywords:** cancer, botanical drug, Huang-Lian (*Coptis chinensis* Franch.), Huang-Qin (*Scutellaria baicalensis* Georgi), Bai-Wei (*Vincetoxicum atratum* (Bunge))

## Abstract

We characterized a so-called “heirloom recipe” Chinese herbal formula (temporarily named Formula X) that contains five Chinese medical botanical drugs, Huang-Lian (*Coptis chinensis* Franch. [Ranunculaceae]), Huang-Qin (*Scutellaria baicalensis* Georgi [Lamiaceae]), Bai-Wei (*Vincetoxicum atratum* (Bunge) C. Morren and Decne. [Apocynaceae]), E-Zhu (*Curcuma aromatica* Salisb. [Zingiberaceae]) and Bai-Zhu (*Atractylodes macrocephala* Koidz. [Asteraceae]). Formula X inhibited the growth of various cancer cells and decreased the expression levels of a panel of proteins, including CD133, Myc, PD-L1, and Slug, in cancer cells. We further found that the inhibition of growth and protein expression were exerted by Huang-Lian, Huang-Qin, and Bai-Wei (formula HHB), which exhibited the same biological effects as those of Formula X. Furthermore, we selected three active chemicals, berberine, baicalin, and saponin from Huang-Lian, Huang-Qin, and Bai-Wei, respectively, to produce a chemical formulation (formula BBS), which exhibited similar effects on cell growth and protein expression as those induced by formula HHB. Both the formulae HHB and BBS suppressed tumor growth in an animal study. Moreover, they decreased the protein levels of Myc and PD-L1 in tumor cells *in vivo*. In summary, we established a novel Chinese herbal formula and a chemical formula that targeted three important processes, tumor maintenance (tumor stem cells), progression, and metastasis, and that influenced the response of tumors to host immunosuppression, for the potentially effective treatment of cancer patients.

## Introduction

Traditional Chinese herbal medicine is widespread in Asia, particularly in Taiwan, China, Japan and Korea. It was developed from a philosophical basis different from that of modern Western medicine. A large number of traditional Chinese medicines (TCM) have still not been fully investigated using modern scientific knowledge. How TCM should be used is controversial at present (Corson and Crews, 2007; Xu and Xia, 2019), and the main concern might be that there is no well-established system to identify and characterize the basis of TCM, such as two “Chi”, “Ying,” and “Yang”, in the human body. Furthermore, unlike pure chemical drugs, whose history, development, and production can be traced and whose biological functions at the cellular and molecular levels have been characterized, Chinese herbal formulae are usually composed of several Chinese botanical drugs; thus, it is difficult to clearly identify the underlying mechanism of their biological effects. However, TCM has been the major medical remedy in Asia for thousands of years and is used in many aspects at present. TCM should be taken as a huge repository of potentially new medical materials and concepts and deserves in-depth exploration.

The fight against cancer has been long. Although we have continuously found new strategies for cancer treatment, their efficacy is restricted, or they have failed due to the genomic instability of cancer cells. Various abnormally mechanisms are developed in cancer cells in response to therapeutic treatment, which eventually cause drug resistance of cancer cells. Multitarget or combinatory molecular targeted therapy might be a more effective cancer treatment (Vasan et al., 2019). Along this line of thinking, Chinese herbal formula, which usually contains several components and potentially affect multiple targets, might be good candidates for cancer treatment. Although investigating how Chinese herbal formulae should be used or fit into modern medicine requires more effort, studying these formulae can at least serve as a starting point for the discovery of potential cancer therapies.

An interesting concept in the theory of TCM is that there are two opposite elements in the human body, Yin-Yang and cold-heat. Among them, the terms heat and cold might not merely represent measurable body temperature and are used to describe two counteracting elements in the human body. Although this concept has not been well elucidated and accepted by modern medical science, these two opposite elements are thought to form a Yin-Yang or cold-heat buffer system to maintain normal physiological conditions. Any intrinsic or extrinsic factors that alter the balance of the buffer systems might cause human disease. In particular, “overheat” inside the body might induce inflammation, and inflammation might increase the level of “heat”. This finding could be interpreted as the heat being like a cellular factor, the level of which is auto-loop increased. Numerous studies have indicated that inflammation is highly associated with the initiation and progression of tumors (Crusz and Balkwill, 2015; Greten and Grivennikov, 2019). Therefore, it is believed in TCM that releasing the “overheat” is expected to effectively prevent or treat tumors.

The botanical drugs Huang-Lian (*Coptis chinensis* Franch. [Ranunculaceae]), Huang-Qin (*Scutellaria baicalensis* Georgi [Lamiaceae]), and Bai-Wei (*Vincetoxicum atratum* (Bunge) C. Morren and Decne. [Apocynaceae]) belong to a family that is classified as having abilities to remove heat and toxins in TCM. A rapidly accumulating number of studies indicates that this family of botanical drugs exerts anticancer effects either alone or with other tumor therapeutic agents. Multiple molecules that these botanical drugs target have been identified to be involved in various oncogenic processes, including cell growth, metastasis, drug resistance and in the response of tumor cells to host immunosuppression (Zhang et al., 2017; Luo et al., 2019).

In addition to the formulae recorded in classic Chinese medical books, many formulae are kept as heirloom recipes and are commonly used in the Chinese community. Although there may be no comprehensively solid evidence to support the function of such formulae, some of them have been used for a long time and are probably helpful for human health. It might be a loss that those formulae will eventually be distorted or lost over time if we view TCM negatively without even performing further characterization. Collecting and testing such formulae in detail is necessary and important for the reasonable usage of Chinese herbal formulae.

We utilized a formula (temporarily named Formula X) comprising five medical botanical drugs, Huang-Lian (HL; *Coptis chinensis* Franch. [Ranunculaceae]), Huang-Qin (HQ; *Scutellaria baicalensis* Georgi [Lamiaceae]), Bai-Wei (BW; *Vincetoxicum atratum* (Bunge) C. Morren and Decne. [Apocynaceae]), E-Zhu (EZ; *Curcuma aromatica* Salisb. [Zingiberaceae]), and Bai-Zhu (BZ; *Atractylodes macrocephala* Koidz. [Asteraceae]). This formula was originally used for anti-fever and anti-inflammation. We tried to identify whether it has an anticancer effect because four components of this formula (HL, HQ, BW, and EZ) have been reported to exert anticancer activity (Ikemoto et al., 2000; Jeon et al., 2011; Wang et al., 2017; Zhang et al., 2017; Gong et al., 2019; Luo et al., 2019; Zhang et al., 2019). In this study, we found that the water extract of Formula X had wide anticancer activity and suppressed the expression of various oncogenic genes, including Her2, estrogen receptor (ER), CD133, Myc, PD-L1 and Slug. We further deconstructed Formula X and then characterized every single component and the various combinations among them, we found that the extract from three components, HL, HQ, and BW together, exhibited the same inhibitory effect on cell growth and gene expression as that from Formula X. By searching previous findings about HL, HQ and BW, three chemicals, berberine, baicalin and saponin, which were used to formulate a new chemical formula (formula BBS), were selected for further studies (Katoh, 2011; Bretones et al., 2015; Stine et al., 2015; Zhang et al., 2017; Zhang et al., 2019). Formula BBS showed the same effect on cell growth and gene expression as that of the herbal formulae. Both the formulae HHB and BBS suppressed tumor growth and the protein expression of Myc and PD-L1 in tumor cells in an animal study.

## Materials and Methods

### Cell Culture and Chemicals

The culture medium (Corning Mediatech, Inc.) and cancer cells used in this study was DMEM for MCF7 (breast cancer) and Huh7 (hepatoma) cells, RPMI1640 for T47D (breast cancer), HCC 1954 (breast cancer), AGS (gastric cancer), LoVo (colon cancer), Mia-Paca2 (pancreatic cancer) and U2OS (osteosarcoma) cells, and DMEM/F12 for MDA-MB231 (breast cancer) cells. The medium was supplemented with 10% fetal bovine serum (FBS). The cells were incubated in a humidified incubator with 5% CO_2_ at 37 °C. The drugs berberine, baicalin and saponin were purchased from Sigma. They were prepared with concentrations of 10 mg/ml as a stock solution and stored in a -20 °C refrigerator.

### Preparation of Botanical Drug Extract

We obtained botanical drugs from commercial Chinese medical herbal stores as ordinary people would. The botanical drugs were imported from China, and they have been regulated and routinely checked by Taiwan’s food and drug administration. The botanical drugs were directly dried materials not subjected to other manufacturing processes. Ten Gram of a single dried botanical drug was added to 100 ml of water (concentration indicated as 100 mg/ml), and then sterilized by high-pressure saturated steam at 121°C for 20 min with an autoclave sterilizer. The supernatants were stored at −20°C. To prepare a formula containing multiple botanical drugs, an equal amount of each herbal extract was mixed. The concentrations indicated in the figures mean the concentration of every single botanical drug in the herbal formula. The identities of the botanical drugs were determined by LC-ESI-MS (detailed method and results are shown in the supplemental method and Supplementary Figure S1) in Lab. The chemical structure of berberine, baicalin and C21 steroidal saponin, the major constituent in the extract from Huang-Lian, Huang-Qin, and Bai-Wei respectively, was derived from the previous studies (Liang et al., 2007; Zhou et al., 2016; Luo et al., 2019) and shown in the Supplementary Figure S2. The samples of each botanical drug were stored for further identification and can be supplied upon request. To maintain the consistency of the biological function across different lots, we further performed a functional assay based on a cytotoxicity assay and Western blotting of specific proteins in each preparation.

### Evaluation of Cell Number

The effect of the botanical drug extract on cell growth was determined by a tetrazolium-based semiautomated colorimetric assay (MTT assay). Cells were seeded at a density of 3,000 cells/well in a 96-well plate. After overnight culture, the cells were treated with various combinations of challenging agents for 4 days as indicated in the figures. Cell numbers were evaluated by the MTT assay with an ELISA reader at OD_540_.

### Western Blotting Analysis

The change in the protein expression profile was determined by Western blotting analysis. After administration of various treatments as indicated in the figures, the cells were lysed with RIPA solution containing a cocktail of protease inhibitors. Aliquots of lysates were subjected to Western blotting analysis. Anti-CD133 and anti-estrogen receptor antibodies were purchased from Santa Cruz Biotechnology. Antibodies against Her2, EGFR, Myc, Slug and PD-L1 were obtained from Cell Signaling Technology. The images were developed with a chemiluminescence reagent.

### Animal Study

To establish the syngeneic mouse model of pancreatic tumors, Pan18 cells (GFPLUC-tagged) derived from pancreatic tumors in EKP (elastase-CreER; LSL-KrasG12D; p53+/−) mice, kindly gifted from Dr. Chia-Ning Shen (Academia Sinica, Taipei, Taiwan), were used.

Female C57BL/6 mice, 6 weeks of age, were obtained from the National Laboratory Animal Breeding and Research Center (Taipei, Taiwan). All animals were housed at the Animal Center of China Medical University (CMU) and maintained in accordance with the institutional animal care protocol. All animal studies were approved by the animal committee of CMU (2017–128). To assess the therapeutic effect of the herbal or chemical formula, tumors were inoculated with 1 × 10^5^ Pan18 cells into the abdominal region of the mouse. After 7 days or 31 days, the mice were orally administered (p.o.) drug a concentration of 50 mg/kg three times a week (once every 2-3 days). The tumor volumes were monitored every 2-3 days after tumor implantation. Tumor sizes were calculated according to the following formula: (length × width^2^)/2.

The lysates of tumor cells were prepared from sacrificed mice and subjected to Western blotting to detect the protein levels of Myc and PD-L1.

### Statistical Test

Results were expressed as the mean ± SD/SEM. Differences between groups were assessed by Student’s t-test. *p* values less than 0.05 were considered significant.

## Results

### Formula X Inhibits Cell Growth and Suppresses Oncogenic Protein Expression in Breast Cancer Cells

**FIGURE 1:**
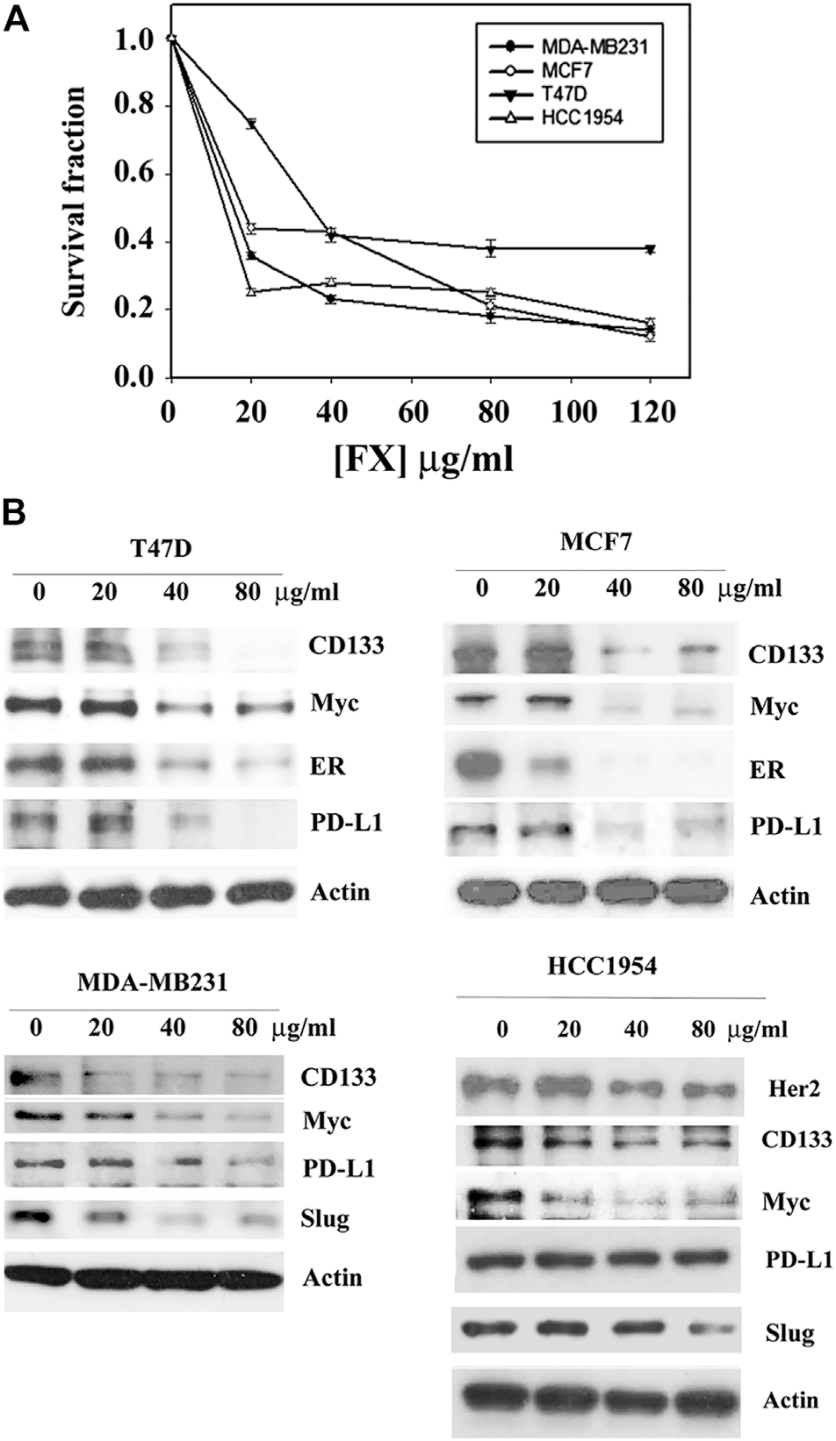
Formula X inhibits cell growth and suppresses oncogenic protein expression in different types of breast cancer cells. **(A)** The indicated cells were treated with a serial dose of Formula X (FX) for 4 days. The level of surviving cells was determined by MTT assay. All data were obtained from at least two independent experiments. The survival fraction showed the fold change relative to the levels of the untreated control. The data are presented as the mean ± SD. **(B)** The cells were treated with various doses of Formula X for 24 h. The lysates were prepared, and the effect of Formula X on the selected protein expression as indicated in the figure was characterized by Western blotting analysis. Actin staining served as a loading control.

The effect of Formula X on cancer cell growth was first determined in breast cancer cells. Four breast cancer cell lines that represent different types of breast cancer were selected for the study. T47D and MCF7 are estrogen receptor (ER)-positive luminal cancer cells, MDA-MB231 is a triple-negative basal cancer cell line, and HCC1954 is a Her2-overexpressing breast cancer cell line. Formula X inhibited the growth of all 4 cell lines in a dose-dependent manner (Figure 1A). Next, we examined the impact of Formula X on the levels of several oncogenic proteins. The Myc protein is an important oncogenic protein that is widely involved in cancer cell growth and progression (Bretones et al., 2015; Stine et al., 2015). The Slug protein is related to cancer cell metastatic ability and might cooperate with Myc to play a critical role in the status of tumor stem cells (Katoh, 2011; Zhou et al., 2019). CD133 is a marker of tumor stem cells (Jang et al., 2017). The PD-L1 protein helps cancer cells escape immunosuppression (Bellmunt et al., 2017). Taken together, we tried to identify the potential effect of Formula X on three important cancer processes, the maintenance, progression and metastasis of cancer, and on the response of cancer cells to the host immune system. The changes in these protein levels under Formula X treatment were determined by Western blotting analysis. As shown in Figure 1B, Formula X decreased the levels of these proteins in a dose-dependent manner in all four breast cancer cell lines, except the level of PD-L1 protein in HCC1954 cells was not affected. In addition, Formula X also suppressed the expression of ER in T47D and MCF7 cells and Her2 in HCC1954 cells. There was no or very little expression of Slug in T47D or MCF7 cells.

### Formula X Inhibits the Growth of a Wide Spectrum of Cancer Cells

**FIGURE 2:**
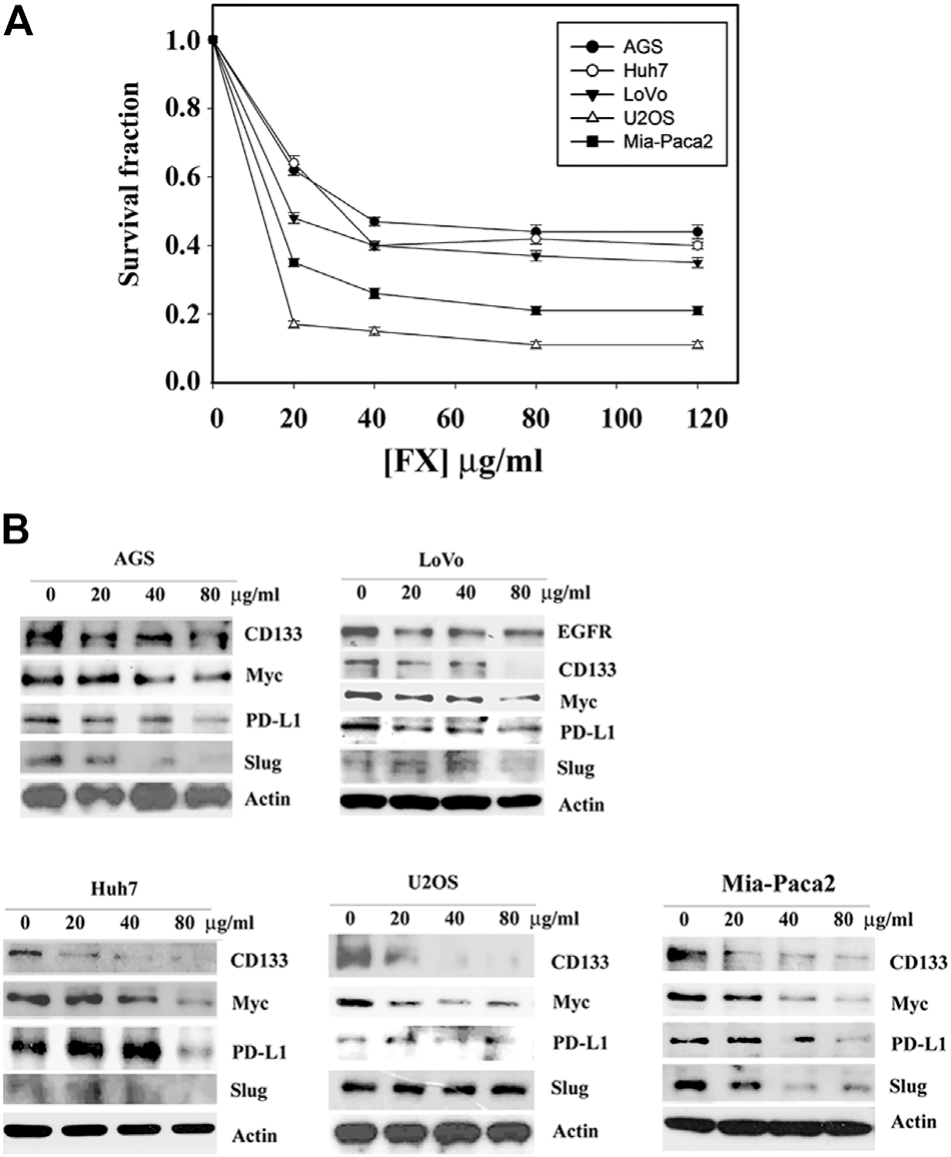
Formula X inhibits cell growth and suppresses oncogenic protein expression in a wide spectrum of cancer cells. **(A)** The indicated cells were treated with a serial dose of Formula X (FX) for 4 days. The level of surviving cells was determined by MTT assay. All data were obtained from at least two independent experiments. The survival fraction showed the fold change relative to the levels of the untreated control. The data are presented as the mean ± SD. **(B)** The cells were treated with various doses of Formula X for 24 h. The lysates were prepared, and the effect of Formula X on the selected protein expression as indicated in the figure was characterized by Western blotting analysis. Actin staining served as a loading control.

We next expanded the study by determining the effect of Formula X on various tissue-original cancer cell lines, including AGS (gastric cancer), LoVo (colon cancer), Huh7 (hepatoma), U2OS (osteosarcoma), and Mia-Paca2 (pancreatic cancer) cells. Formula X inhibited the growth of all cancer cells tested (Figure 2A). Consistent with the results from Formula X-treated breast cancer cell lines, Formula X decreased the level of selected proteins in all cancer cells tested, except the Slug protein level of U2OS cells was not decreased by Formula X (Figure 2B). Because epidermal growth factor receptor (EGFR) is a critical target for colon cancer therapy (Tamas et al., 2015), the change in EGFR expression under Formula X treatment was also examined. Formula X decreased the protein level of EGFR in LoVo cells (Figure 2B). The staining of Slug in Huh7 and LoVo cells and the staining of PD-L1 in U2OS cells were very weak, which might be due to their low expression in these cells (Figure 2B), and therefore, it is hard to determine the changes of those proteins under Formula X treatment.

### Inducibility of Drug Resistance to Formula X in Cancer Cells

**FIGURE 3:**
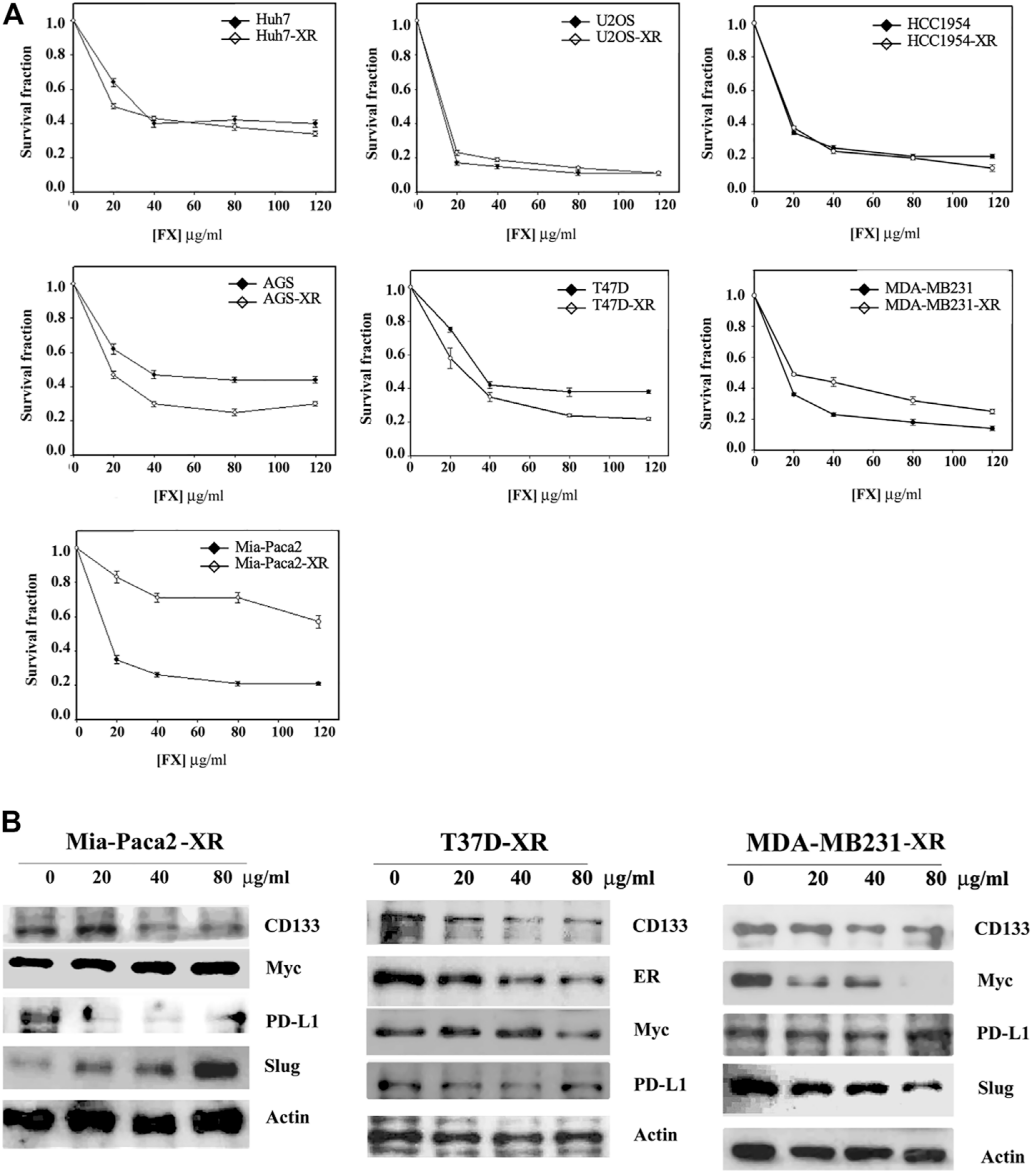
Determination of inducible drug resistance by Formula X treatment. The cells were treated with 40 μg/ml Formula X (FX) for 24 h and then released for another 24 h (-XR cells). Subsequently, the XR cells were treated with a serial dose of Formula X for 4 days. The level of surviving cells was determined by MTT assay. For direct comparison, the curve of untreated cells was redrawn from Figures 1A, 2A. The data are shown as the mean ± SD. **(B)** The XR cells manipulated from Mia-Paca2, T47D and MDA-MB231 cells were treated with a serial dose of Formula X. Western blotting analysis showed the change in protein expression in these cells.

Cancer therapy often fails due to the development of drug resistance. Experimentally, the drug resistance of cancer cells can be induced by short-term treatment of cancer cells with a nonlethal dosage of anticancer drugs (Yeh et al., 2002). We then tried to identify whether drug resistance to Formula X would be induced in cancer cells. The cancer cells indicated in Figure 3A were treated with 40 μg/ml Formula X for 24 h and then released for another 24 h. The sensitivity of these cells (-XR cells) to Formula X was evaluated. As shown in Figure 3A, no change in sensitivity was observed in Huh7-XR, U2OS-XR or HCC1954-XR cancer cells; a slightly more sensitive response was observed in AGS-XR and T47D-XR cancer cells; and slightly more resistance was observed in MDA-MB231-XR cancer cells. In contrast, a dramatic increase in resistance was induced in Mia-Paca2-XR cancer cells.

Western blotting analysis showed that Formula X decreased the protein levels of CD133 and PD-L1 but increased the protein levels of Myc and Slug in Mia-Paca2-XR cells (Figure 3B). In contrast, Formula X still suppressed the protein levels of Myc and Slug in T47D and MDA-MB231-XR cells (Figure 3B). In addition, the expression levels of PD-L1 in T47D-XR and MDA-MB231-XR cells were not suppressed by Formula X, suggesting that PD-L1 might not be involved in the sensitivity of these cancer cells to Formula X.

### Deconstruction of Formula X

**FIGURE 4:**
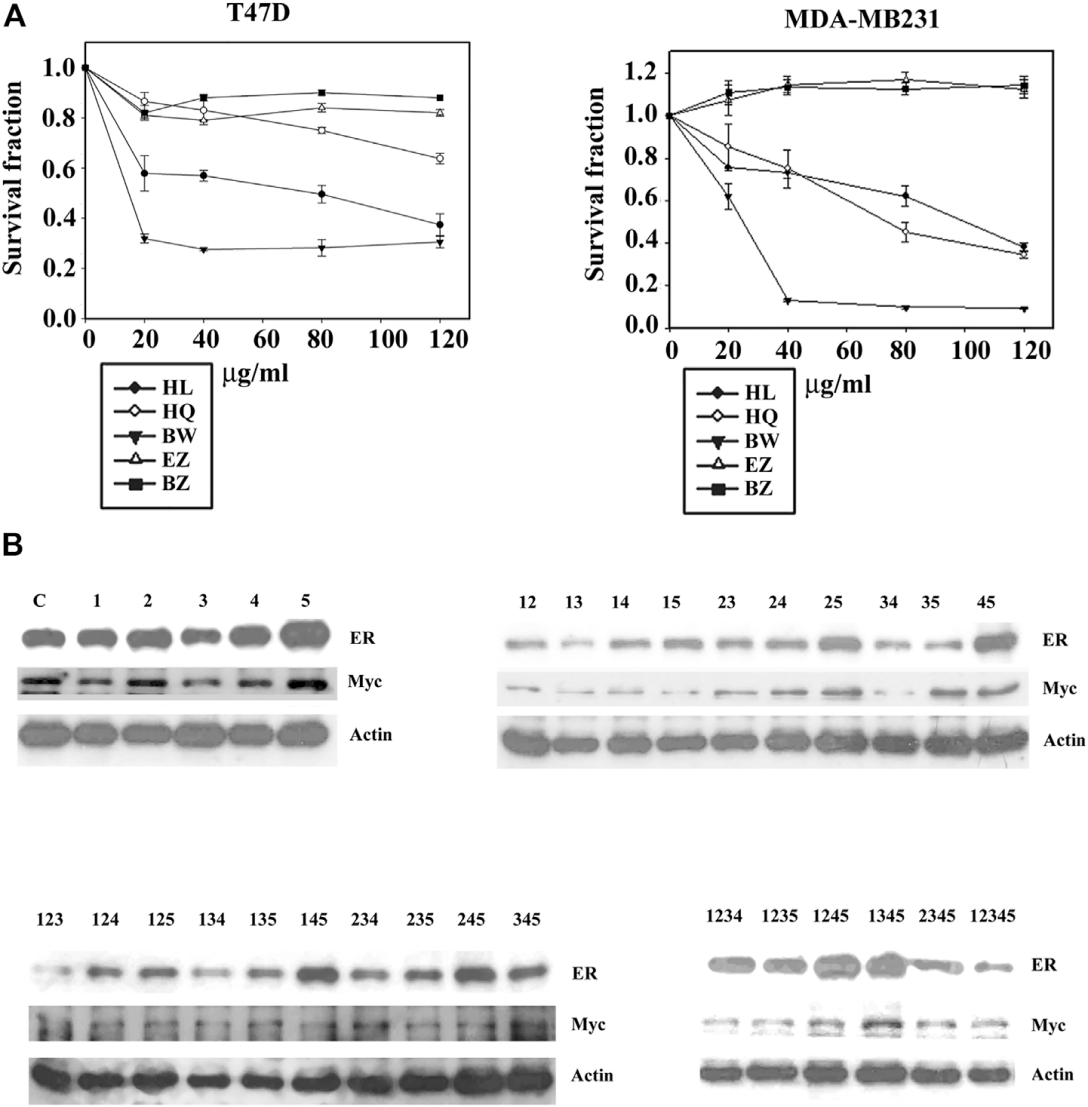
Deconstruction of Formula X. **(A)** T47D and MDA-MB231 cells were treated with various doses of each single component of Formula X (FX) for 4 days. The level of surviving cells was determined by MTT assay. The survival fraction showed the fold change relative to the levels observed in the untreated control. The data are presented as the mean ± SD. **(B)** T47D cells were treated with 40 μg/ml of each single or all possible combinations among the five components of Formula X for 24 h. Western blotting analysis was used to characterize the effect of each treatment on the level of the indicated protein. 1) HL, 2) HQ, 3) BW, 4) EZ, 5) BZ.

To further identify the function of each component of Formula X, the sensitivity of T47D and MDA-MB231 cells to each single drug of Formula X was determined. As shown in Figure 4A, BW had the highest inhibitory effect on both cell lines. HL and HQ showed moderate inhibitory effects. However, EZ and BZ had no inhibitory effect and even slightly increased the growth of MDA-MB231 cells.

We then decided to fully deconstruct Formula X by determining the level of growth inhibition induced by each single component and all possible combinations among them in T47D cells. The data are shown in Table 1. The results showed that HL and BW were the major components exerting inhibition of growth. HQ exhibited a smaller inhibitory activity; in contrast, both EZ and BZ had no effect on T47D cell growth. All the combinations containing HL and/or BW showed a similar level of growth inhibition; for example, the combinations of HL/BW, HQ/BW, HL/HQ/BW and HL/HQ/BW/BZ exhibited comparable inhibitory effects to that of the whole Formula X (the survival fractions were 0.37, 0.34, 0.37, 0.33, and 0.40, respectively).

**TABLE 1:** T47D cells were seeded at a density of 2000 cells/well in 96-well plates. The cells were treated with a single drugs as indicated or various combinations of drugs at 40 μg/ml for 4 days. The number of survival fractions is shown as the fold change relative to levels observed in the untreated control. The results are presented as the mean ± SD. 1) HL, 2) HQ, 3) BW, 4) EZ, 5) BZ.

Treatment	1 (HL)	2 (HQ)		3 (BW)	4 (EZ)	5 (BZ)					
Survival fraction	0.57 ± 0.03	0.83 ± 0.02		0.39 ± 0.04	1.17 ± 0.01	1.03 ± 0.02					
Treatment	1 + 2		1 + 3	1 + 4	1 + 5	2 + 3	2 + 4	2 + 5	3 + 4	3 + 5	4 + 5
Survival fraction	0.62 ± 0.03		0.37 ± 0.01	0.83 ± 0.07	0.78 ± 0.02	0.34 ± 0.04	1.00 ± 0.01	0.88 ± 0.01	0.54 ± 0.07	0.49 ± 0.04	0.90 ± 0.04
Treatment	1 + 2 + 3		1 + 2 + 4	1 + 2 + 5	1 + 3 + 4	1 + 3 + 5	1 + 4 + 5	2 + 3 + 4	2 + 3 + 5	2 + 4 + 5	3 + 4 +5
Survival fraction	0.37 ± 0.04		0.89 ± 0.03	0.82 ± 0.01	0.42 ± 0.02	0.49 ± 0.02	0.82 ± 0.02	0.50 ± 0.03	0.49 ± 0.04	1.02 ± 0.09	0.52 ± 0.05
Treatment	1 + 2 + 3 + 4		1 + 2 + 3 + 5	1 + 2 + 4 + 5	1 + 3 + 4 + 5	2 + 3 + 4 + 5					
Survival fraction	0.46 ± 0.09		0.33 ± 0.01	0.97 ± 0.01	0.43 ± 0.01	0.51 ± 0.08					
Treatment	1 + 2 + 3 + 4 +5										
Survival fraction	0.40 ± 0.02										

The effect of all kinds of combinations of components on protein expression was characterized on the basis of the change in ER and Myc in T47D cells to assess their potential function in cancer cell growth. The results showed that the combinations containing HL and/or BW could effectively suppress ER and Myc expression; in contrast, BZ alone or combined with other components increased protein expression and particularly the level of ER expression (Figure 4B). Of all ten combinations of the three components, all combinations containing BZ increased ER expression. The highest level of ER was observed in HL/EZ/BZ- and HQ/EZ/BZ-treated T47D cells, and the lowest level of ER was observed in HL/HQ/BW-treated cells.

**FIGURE 5:**
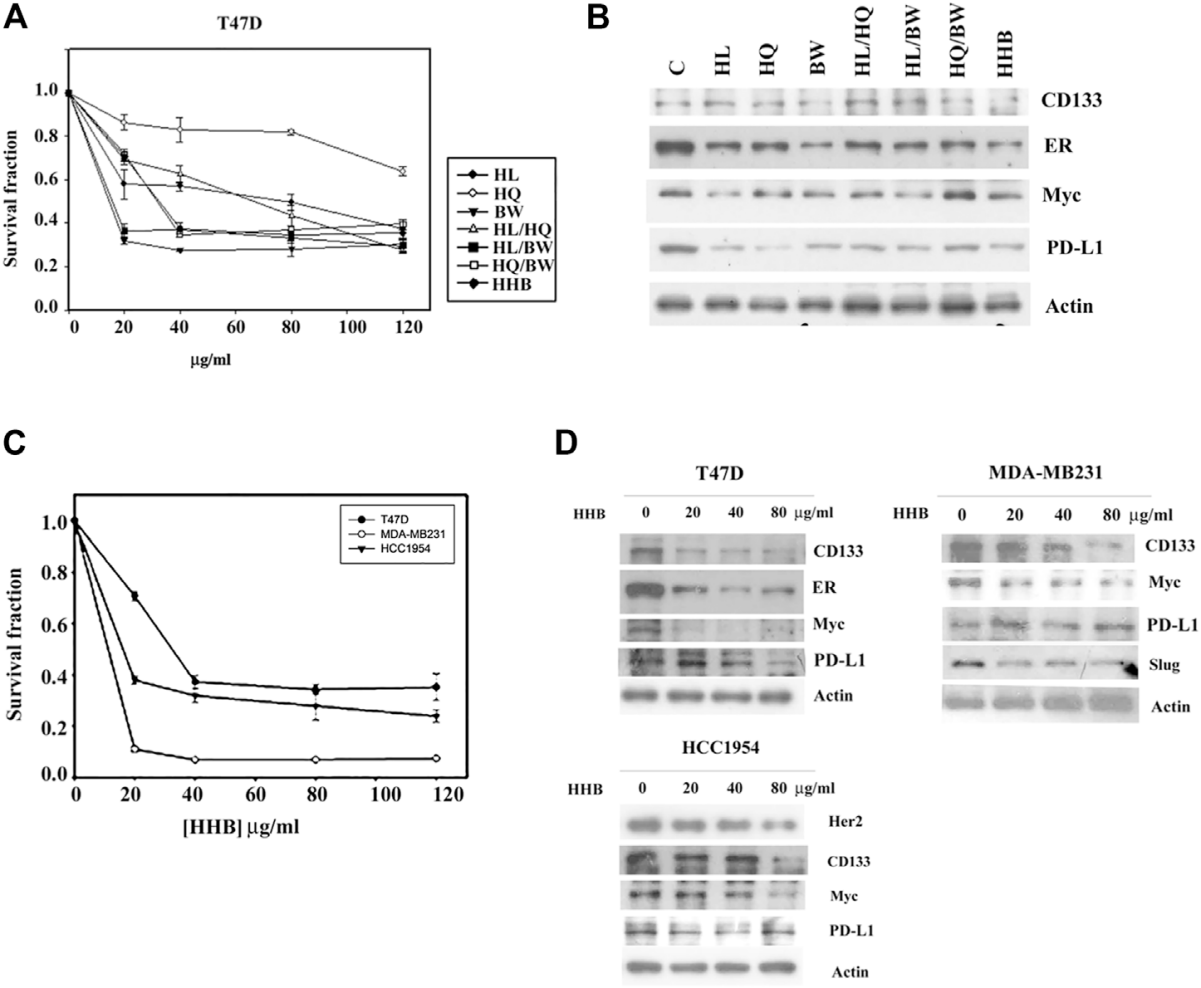
The biological effect of the formula HHB. **(A)** T47D cells were treated with various doses of HL, HQ and BW or all possible combinations for 4 days. The level of surviving cells was determined by MTT assay. Formula HHB is a combination treatment with HL, HQ and BW. **(B)** T47D cells were treated with 40 μg/ml of each single or all possible combinations of the indicated agents for 24 h. Western blotting analysis was performed to characterize the change in the protein expression profile. Three breast cancer cell lines, T47D, MDA-MB231 and HCC 1954, were treated with various doses of the formula HHB. The effect of the formula HHB on cell growth was determined by MTT assay **(C)**, and the effect on protein expression was characterized by Western blotting analysis **(D)**. The survival fraction showed the fold change relative to the levels observed in the untreated control. The data are presented as the mean ± SD.

We then removed EZ and BZ from Formula X. The biological effect of the new formula (HHB) was characterized using T47D cells as an assay system. As shown in Figure 5A, both single components and all possible combinations among these three components suppressed cell growth. Western blotting analysis showed that all treatments decreased the levels of proteins, including CD133, ER, Myc and PD-L1, to different degrees, and HHB suppressed the levels of all proteins to their lowest levels (Figure 5B). To further confirm this result, three breast cancer cell lines, T47D, MDA-MB231 and HCC 1954, were treated with a dose course of HHB. The formula HHB suppressed the growth of all three cancer cell lines in a dose-dependent manner (Figure 5C). MDA-MA231 cells were the most sensitive to the formula HHB. Western blotting analysis showed that the formula HHB decreased the expression of selected proteins in all three breast cancer cell lines, except the expression of PD-L1 was not affected by the formula HHB in MDA-MB231 cells (Figure 5D).

### Establishment of a Chemical Formula

**FIGURE 6:**
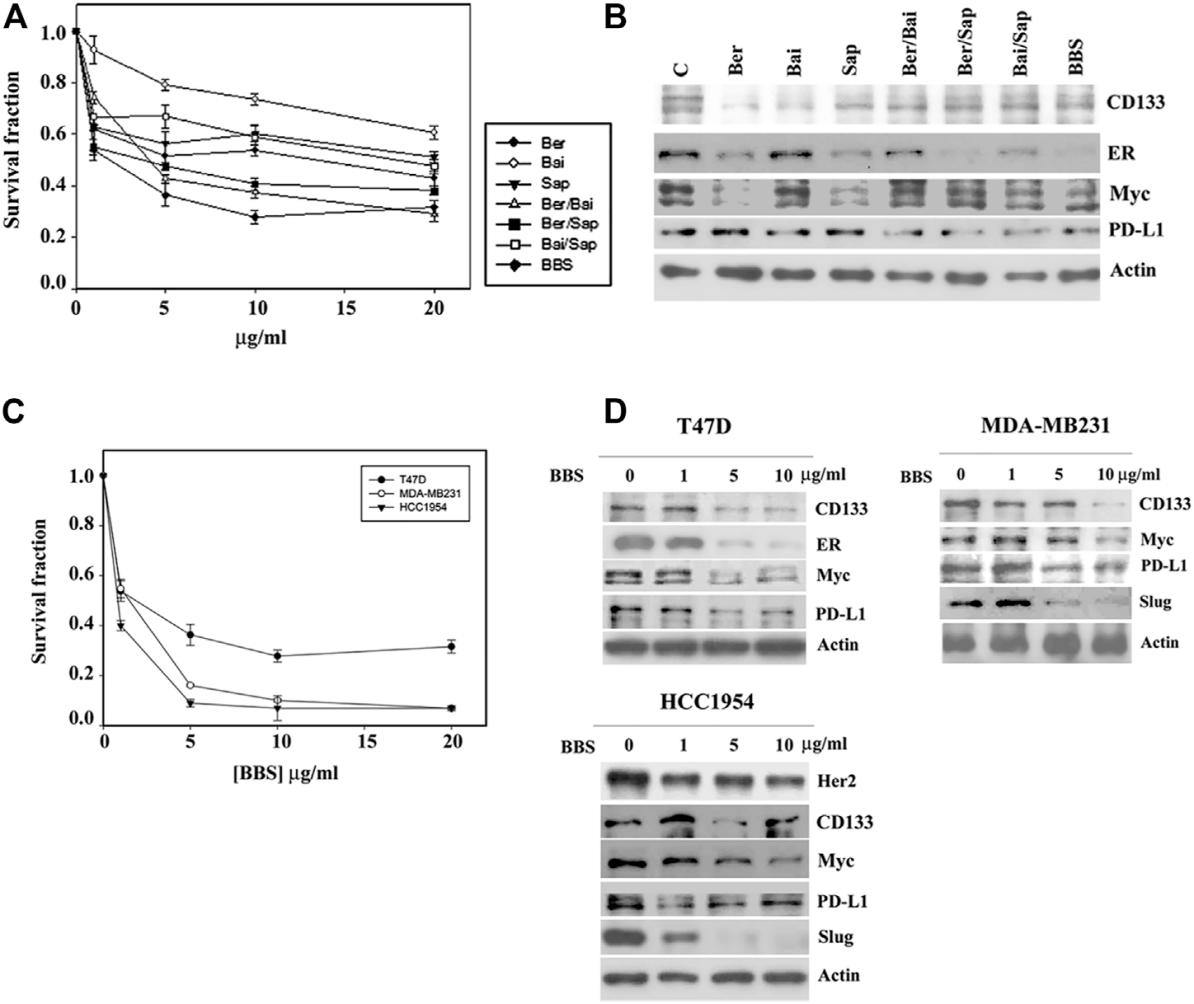
The biological effect of the BBS formula. **(A)** T47D cells were treated with various doses of berberine (Ber), baicalin (Bai) and saponin (Sap) or all possible combinations for 4 days. The level of surviving cells was determined by MTT assay. Formula BBS is a combination treatment with berberine (Ber), baicalin (Bai), and saponin (Sap). **(B)** T47D cells were treated with 5 μg/ml of each single or all possible combinations of the indicated agents for 24 h. Western blotting analysis was performed to characterize the change in the protein expression profile. Three breast cancer cell lines, T47D, MDA-MB231 and HCC 1954, were treated with various doses of the BBS formula. The effect of the BBS formula on cell growth was determined by MTT assay **(C)**, and protein expression was characterized by Western blotting analysis **(D)**. The survival fraction showed the fold change relative to the levels observed in the untreated control. The data are presented as the mean ± SD.

The quality control of medical botanical drugs is always an important issue because the botanical drug content might be different with changes in environmental conditions. Physicochemical methods might be used to identify the botanical drug but might not be able to fully determine the biological and/or medical effect of the botanical drug. To set up a functional assay system to control the quality of botanical drugs, in this study, we used a cytotoxic assay and Western blotting of Myc to assess that the consistency of every preparation had been maintained. On the other hand, much effort has been made to identify the principle effective compound in medical botanical drugs and to discover the many related effective chemical compounds of specific medical botanical drugs (Tan et al., 2011; Cai et al., 2018; Luo et al., 2019; Wang et al., 2019). We searched the previous findings about HL, HQ and BW (Liang et al., 2007; Zheng et al., 2007; Xu et al., 2016; Mortazavi et al., 2020; Orzechowska et al., 2020) and selected three chemicals, berberine, baicalin and saponin, to form a chemical formula (formula BBS) for further studies. Among them, C21 steroidal saponin was found to be a major component in BW; however, it was commercially unavailable. A mixture of saponins (S4521) obtained from Sigma was used, which might be expected to have similar biological characteristics to those of C21 steroidal saponins. Consistent with the effect of the formula HHB, both a single agent and different combinations among three chemicals inhibited the growth of T47D cells, and the three chemicals together (formula BBS) achieved maximal growth inhibition (Figure 6A). Western blotting analysis showed that all treatments exerted different effects on the protein levels of CD133, ER, Myc and PD-L1, while the formula BBS suppressed the expression levels of all four proteins (Figure 6B).

Furthermore, a serial concentration of the formula BBS was used to treat three breast cancer cell lines, T47D, MDA-MB231 and HCC 1954. The formula BBS exhibited a dose-dependent effect of growth inhibition in all three cancer cell lines. MDA-MB231 and HCC1954 cells showed similar sensitivities to the BBS formula, and T47D cells exhibited a higher resistance to the BBS formula (Figure 6C). Western blotting analysis indicated that the BBS formula suppressed protein expression in these three cancer cell lines in a pattern similar to that of the HHB formula (Figure 6D and compared to Figure 5D). Our results suggested that the BBS formula could at least partly replace the HHB formula on the basis of its effects on breast cancer cells.

### Formulae HHB and BBS do Not Induce Drug Resistance in Mia-Paca2 Cells

**FIGURE 7:**
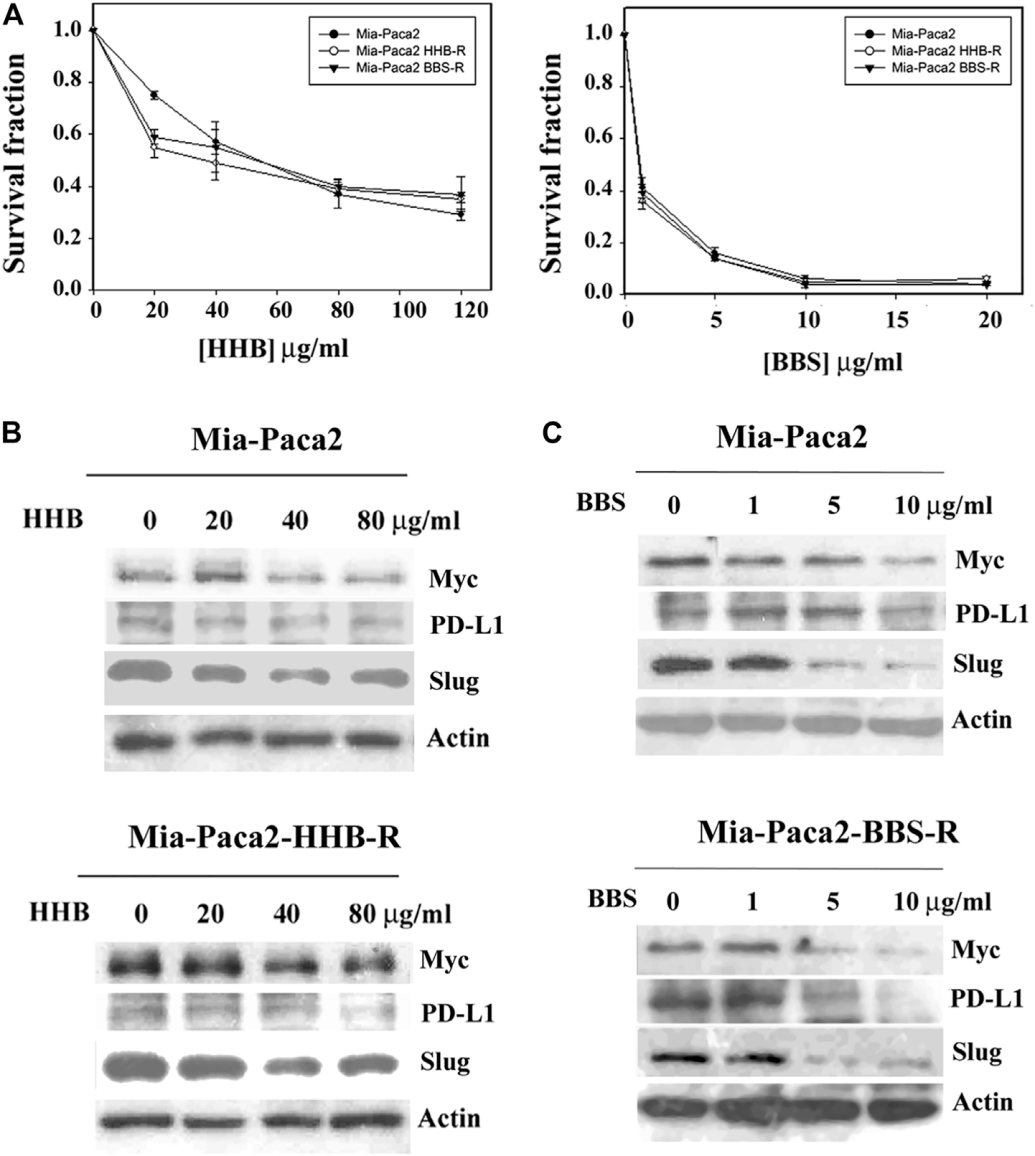
Determination of inducible drug resistance by formula HHB and BBS treatment. **(A)** Mia-Paca2 cells were treated with 40 μg/ml formula HHB or 5 μg/ml formula BBS for 24 h and then released for another 24 h (Mia-Paca2 HHB-R cells and Mia-Paca2 BBS-R cells, respectively). Subsequently, the HHB-R/BBS-R cells were treated with a serial dose of HHB/BBS for 4 days. The level of surviving cells was determined by MTT assay. The survival fraction shows the fold change relative to the levels observed in the untreated control. The data are presented as the mean ± SD. The cells shown in the figure were treated with a serial dose of the formula HHB **(B)** or the formula BBS **(C)** for 24 h. The changes in Myc, PD-L1 and Slug expression under the indicated treatments were characterized by Western blotting analysis.

A previous experiment showed that Formula X induced drug resistance in Mia-Paca2 cells (Figure 3A). Whether the formulae HHB and BBS also induce drug resistance in Mia-Paca2 cells was determined. Mia-Paca2 cells were treated with 40 μg/ml formula HHB or 5 μg/ml formula BBS for 24 h and then released for another 24 h (Mia-Paca2-HHB-R and Mia-Paca2-BBS-R cells, respectively). The effect of the formula HHB or BBS on growth inhibition in these manipulated cells was evaluated. As shown in Figure 7A, neither HHB nor BBS induced drug resistance in Mia-Paca2 cells. Consistently, Myc, PD-L1 and Slug expression was suppressed in untreated and both HHB-R and BBS-R Mia-Paca2 cells (Figures 7B,C).

### Both Formulae HHB and BBS Exhibited Inhibitory Effects on Tumor Growth and Myc and PD-L1 Expression in Animal Studies

**FIGURE 8:**
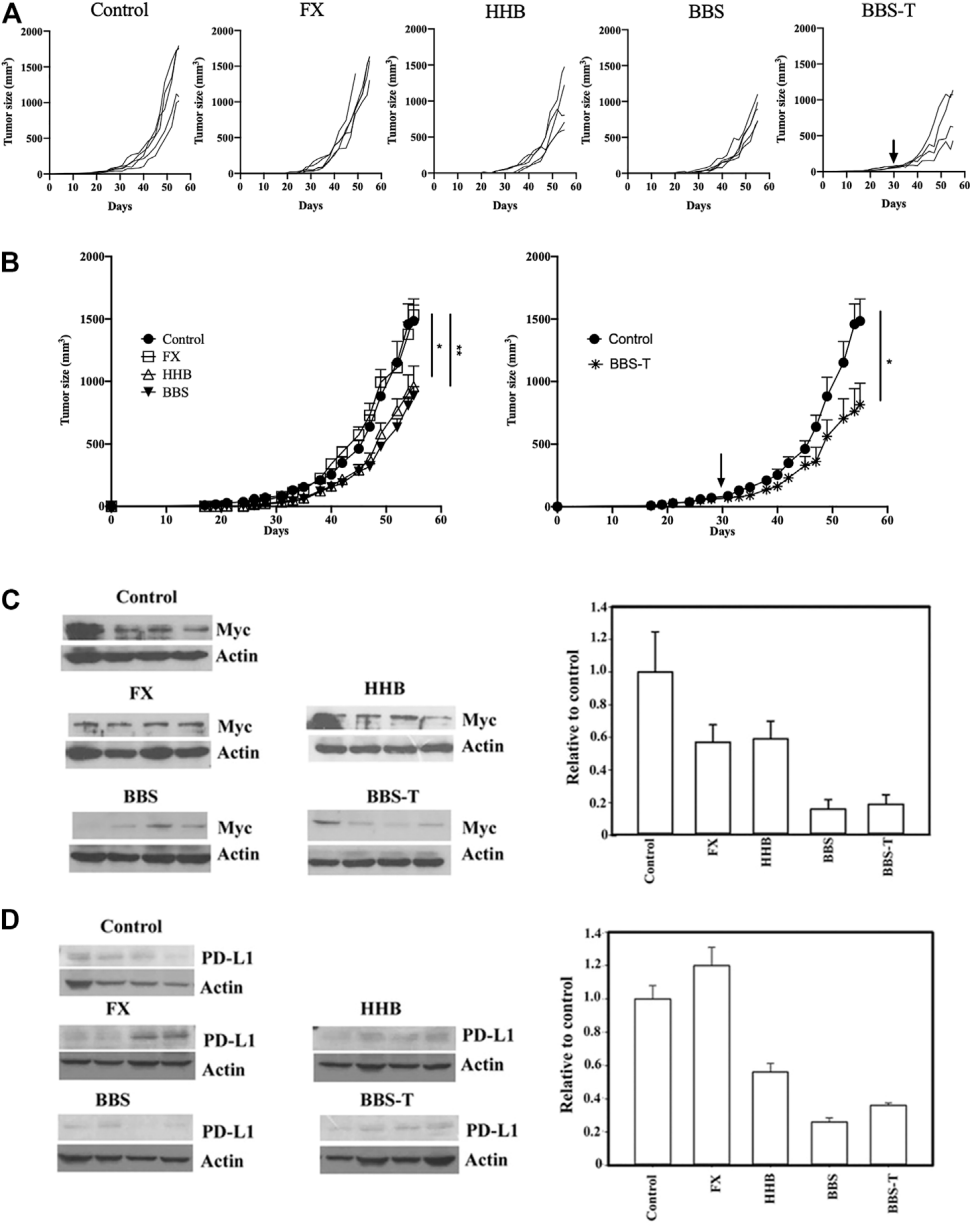
The antitumor effect of formula FX/HHB/BBS treatment. **(A)** Pan18 tumor-bearing mice (4-5 animals/group) were orally administered ddH_2_O (control), Formula X (FX), HHB, or BBS on Day 7 or orally administered formula BBS on Day 31 (to detect potential therapeutic effects; BBS-T). Each curve represents each individual mouse. **(B)** The results were pooled and are expressed as the means ± SEMs. The tumor diameters are shown (mm^3^). *p < 0.05, **p < 0.01. The lysates from the tumors of each sacrificed mouse were subjected to Western blotting to detect the protein levels of Myc **(C)** and PD-L1 **(D)**. No particular region of the image was solely manipulated. The density of each band was determined by an internet available program (ImageJ) and normalized to the density of the actin band. The data are expressed as the fold change relative to the levels observed in the control ±SD (right panel of Figures C and D).

Since these formulae decreased the protein level of PD-L1 in cancer cells, a mouse pancreatic cancer cell, Pan18, implanted mouse was used as an immunocompetent model system to assay drug inhibitory effects on tumor growth in animals. As shown in Figures 8A,B, while Formula X did not suppress tumor growth, formulae HHB and BBS decreased the rate of tumor growth. To evaluate the potential therapeutic effect of formula BBS, a group of Pan18-implanted mice was treated with formula BBS starting at 31 days after implantation. The formula BBS decreased the growth rate of tumors, suggesting that the formula BBS is a potentially effective therapeutic formula (Figure 8B; BBS-T).

To identify whether these formulae could affect the expression of protein in the tumor-bearing mice as was observed in cultured cells, the levels of Myc and PD-L1 were detected in all tumors from the scarified mice. As shown in Figure 8C, all treatments decreased the expression of Myc to different degrees. The protein level of Myc was reduced to half of the level of the control group in Formula X- and HHB-treated groups and reduced to below 20% of the level of the control group in the formula BBS-treated group. Formula X did not suppress, or even slightly increase, the protein level of PD-L1, whereas all other treatments decreased the protein level of PD-L1 to a similar degree as they did for Myc expression (Figure 8D).

## Discussion

In this study, we deconstructed and characterized each component of the Chinese herbal Formula X. Interestingly, we found that two components (EZ and BZ) of Formula X exhibited effects opposite to those of the other three components, while they did not influence the biological effects of Formula X in cultured cells. According to the description regarding the functions of EZ and BZ in TCM (the information can be obtained from the websites en. wikibooks.org/wiki/Traditional Chinese Medicine (in English), and cht.a-hospital.com (in Chinese)), both have functions in the stomach and pancreatic meridians, which means they may protect and enhance the function of the digestive system, or in other words, they may influence nutrient uptake. This finding might represent the philosophy of TCM. The formula used in TCM is usually based on the role, so called “jun, chen, zuo and shi” (Wu et al., 2014), in which some components are principle effective components and the others are helpers to increase the effect or decrease the side effects induced by major components or to enhance the speed of drug delivery in the body.

However, the results of the animal study indicated that Formula X did not inhibit tumor growth in animals. In contrast, both formulae HHB and BBS reduced tumor growth in animals. This result suggested that EZ and BZ might reverse the inhibitory effects of HL, HQ and BW on tumor growth in animals, although Formula X showed a similar effect of growth inhibition as those of formulae HHB and BBS in the culture-cell study. This finding raises the possibility that the metabolic products of EZ and BZ in animals might cause some uncharacterized processes to occur that reverses the effect of the other three components of Formula X. Furthermore, Western blotting analysis demonstrated that Formula X did not decrease the expression of PD-L1 but still reduced the protein level of Myc. This result suggested that immuno-suppression of tumor growth might play a critical role and that EZ and BZ could block the immune response of host animals by restoring the level of PD-L1 in tumor cells. This phenomenon probably could not be observed when using immunodeficient mice as the study system. In addition, whether the finding of no effect of Formula X on tumor growth in animals is specific to a particular type of tumor cell or is a common phenomenon remains to be identified.

Berberine has been reported to inhibit cancer cell growth and metastasis, and exhibit anti-inflammatory properties (Wang et al., 2020). Similarly, baicalin (Verma et al., 2021) and saponin (Elekofehinti et al., 2021) are also reported to be involved in multiple processes of cancer cells, including cell growth and metastasis. The underlying mechanism is not well-identified how a single molecule such as berberine, baicalin, or saponin could affect such many biological processes. In this study, we showed all three formulae affected the expression levels of a panel of proteins, including growth factor and steroid receptors, oncogenic proteins, tumor stem cell-related proteins, and immune response-related proteins, in various cancer cells. It is reasonable that these formulae might target multiple molecules and signaling pathways, but the possibility that they target a “master” molecule in the signal transduction network could not be ruled out. For example, the Myc protein was identified to regulate the expression of PD-L1 and CD133 (Gopisetty et al., 2013; Casey et al., 2016).

The primary purpose of this study is to characterize a Chinese herbal formula through deconstruction and reconstitution of the formula. Our results transformed the original formula (Formula X) into a new formula (Formula HHB), and rationally combined three chemicals to establish a novel chemical formula (Formula BBS), which might be more suitable for modern medicine to potentially treat cancer. In this study, the changes in protein expression under treatment might be served as molecular markers to determine the effect of the individual or combined component. However, the underlying mechanism of both herbal and chemical formulae has not been characterized. To identify the direct target or the signaling transduction pathway is important and necessary for further understanding and developing both herbal and chemical formulae. We indeed try to identify and characterize the underlying mechanism of the effect of both Formula HHB and Formula BBS in a single type of cancer by modern biochemical and molecular techniques and expect to deeper elucidate the function of both formulae.

We observed that the formulae HHB and BBS suppressed Myc expression and did not induce drug resistance in pretreated Mia-Paca2 cells. This result indicated that EZ and BZ might be involved in the induction of drug resistance by increasing Myc protein expression when compared with the result from Formula X pretreated Mia-Paca2 cells (Mia-Paca2-XR).

Western blot analysis showed that every single component of formula HHB reduced level of selected protein to a different degree. The inhibitory effect of HL and HQ on PD-L1 expression, BW on CD133 and ER expression, and HL on Myc expression was higher than that of other compounds (Figure 5B). This result might represent the potential advantage benefit of traditional Chinese herbal medicine, in which the combined different botanical drugs cooperatively affect multiple targets. Similar results were also observed in the chemical formula BBS (Figure 6B). Our results suggested that deconstruction and reconstitution of a Chinese herbal formula could further understand the possible underlying mechanism of its modern biological function. Importantly, we can establish a new chemical formula with a rational combination of different chemical drugs.

Comparing the doses of the formulae HHB and BBS used, 40 μg/ml formula HHB and 5 μg/ml formula BBS were needed to reduce T47D cell growth to 40% of the control level. The levels of the three chemicals (berberine, baicalin and saponin) in the formula BBS were generally below 10% of the content of the corresponding botanical drugs (HL, HQ and BW) in the formula HHB, and the efficiency of extraction should not be 100%; therefore, the amount of these three chemicals in the extract from 40 μg/ml formula HHB was theoretically below 5 μg/ml. This suggests that the inhibitory effect of the formula HHB on tumor growth might not be totally caused by these three chemicals. In an animal study, both formulae HHB and BBS suppressed tumor growth to a similar degree, while the formula BBS decreased Myc and PD-L1 levels to a much lower level than the formula HHB did. Taken together, these findings suggest that some uncharacterized components in the extract of the formula HHB exerted effects of growth inhibition through PD-L1- and Myc-independent pathways.

Furthermore, we demonstrated that these herbal formulae decreased the protein levels of PD-L1 and several other important oncogenic proteins. This raises the possibility that they might be combined with conventional cancer therapies, such as immuno- or chemotherapy. In particular, they suppressed the protein levels of Her2, estrogen receptor and Myc in different types of breast cancer cells, suggesting that they might be broadly effective in breast cancer, although this possibility remains to be further characterized.

Formula X was originally used to treat fever, and no record indicates that it has anticancer activity. Our study indeed suggests that Formula X might not be suitable for cancer treatment, partly because it increases PD-L1 expression and consequently reduces the host immunosuppression of tumor growth. However, through thoughtful characterization, we transformed it into an effective novel herbal formula. We believe this approach provides a method to renew and understand traditional Chinese medicine.

## Data Availability

The raw data supporting the conclusion of this article will be made available by the authors, without undue reservation.

## References

[B1] Bellmunt J. Powles T. Vogelzang N. J. (2017). A Review on the Evolution of PD-1/pd-L1 Immunotherapy for Bladder Cancer: The Future Is Now. Cancer Treat. Rev. 54, 58–67. 10.1016/j.ctrv.2017.01.007 28214651

[B2] Bretones G. Delgado M. D. León J. (2015). Myc and Cell Cycle Control. Biochim. Biophys. Acta 1849 (5), 506–516. 10.1016/j.bbagrm.2014.03.013 24704206

[B3] Cai F. F. Zhou W. J. Wu R. Su S. B. (2018). Systems Biology Approaches in the Study of Chinese Herbal Formulae. Chin. Med. 13, 65. 10.1186/s13020-018-0221-x 30619503 PMC6311004

[B4] Casey S. C. Tong L. Li Y. Do R. Walz S. Fitzgerald K. N. (2016). Erratum for the Report "MYC Regulates the Antitumor Immune Response through CD47 and PD-L1" by S. C. Casey, L. Tong, Y. Li, R. Do, S. Walz, K. N. Fitzgerald, A. M. Gouw, V. Baylot, I. Gütgemann, M. Eilers, D. W. Felsher. Science 352 (6282), 227–231. 10.1126/science.aac993510.1126/science.aaf7984 26966191 PMC4940030

[B5] Corson T. W. Crews C. M. (2007). Molecular Understanding and Modern Application of Traditional Medicines: Triumphs and Trials. Cell 130 (5), 769–774. 10.1016/j.cell.2007.08.021 17803898 PMC2507744

[B6] Crusz S. M. Balkwill F. R. (2015). Inflammation and Cancer: Advances and New Agents. Nat. Rev. Clin. Oncol. 12 (10), 584–596. 10.1038/nrclinonc.2015.105 26122183

[B7] Elekofehinti O. O. Iwaloye O. Olawale F. Ariyo E. O. (2021). Saponins in Cancer Treatment: Current Progress and Future Prospects. Pathophysiology 28 (2), 250–272. 10.3390/pathophysiology28020017 35366261 PMC8830467

[B8] Gong B. Kao Y. Zhang C. Zhao H. Sun F. Gong Z. (2019). Exploring the Pharmacological Mechanism of the Herb Pair "HuangLian-GanJiang" against Colorectal Cancer Based on Network Pharmacology. Evid. Based Complement. Altern. Med. 2019, 2735050. 10.1155/2019/2735050 PMC6906823 31871473

[B9] Gopisetty G. Xu J. Sampath D. Colman H. Puduvalli V. K. (2013). Epigenetic Regulation of CD133/PROM1 Expression in Glioma Stem Cells by Sp1/myc and Promoter Methylation. Oncogene 32 (26), 3119–3129. 10.1038/onc.2012.331 22945648 PMC3820114

[B10] Greten F. R. Grivennikov S. I. (2019). Inflammation and Cancer: Triggers, Mechanisms, and Consequences. Immunity 51 (1), 27–41. 10.1016/j.immuni.2019.06.025 31315034 PMC6831096

[B11] Ikemoto S. Sugimura K. Yoshida N. Yasumoto R. Wada S. Yamamoto K. (2000). Antitumor Effects of Scutellariae Radix and its Components Baicalein, Baicalin, and Wogonin on Bladder Cancer Cell Lines. Urology 55 (6), 951–955. 10.1016/s0090-4295(00)00467-2 10840124

[B12] Jang J. W. Song Y. Kim S. H. Kim J. Seo H. R. (2017). Potential Mechanisms of CD133 in Cancer Stem Cells. Life Sci. 184, 25–29. 10.1016/j.lfs.2017.07.008 28697984

[B13] Jeon J. Park K. A. Lee H. Shin S. Zhang T. Won M. (2011). Water Extract of Cynanchi Atrati Radix Regulates Inflammation and Apoptotic Cell Death through Suppression of IKK-Mediated NF-Κb Signaling. J. Ethnopharmacol. 137 (1), 626–634. 10.1016/j.jep.2011.06.022 21718772

[B14] Katoh M. (2011). Network of WNT and Other Regulatory Signaling Cascades in Pluripotent Stem Cells and Cancer Stem Cells. Curr. Pharm. Biotechnol. 12 (2), 160–170. 10.2174/138920111794295710 21044011

[B15] Liang M. Zheng Z. Yuan Y. Kong L. Shen Y. Liu R. (2007). Identification and Quantification of C21 Steroidal Saponins from Radix Cynanchi Atrati by High-Performance Liquid Chromatography with Evaporative Light Scattering Detection and Electrospray Mass Spectrometric Detection. Phytochem. Anal. 18 (5), 428–435. 10.1002/pca.998 17624893

[B16] Luo H. Vong C. T. Chen H. Gao Y. Lyu P. Qiu L. (2019). Naturally Occurring Anti-cancer Compounds: Shining from Chinese Herbal Medicine. Chin. Med. 14, 48. 10.1186/s13020-019-0270-9 31719837 PMC6836491

[B17] Mortazavi H. Nikfar B. Esmaeili S. A. Rafieenia F. Saburi E. Chaichian S. (2020). Potential Cytotoxic and Anti-metastatic Effects of Berberine on Gynaecological Cancers with Drug-Associated Resistance. Eur. J. Med. Chem. 187, 111951. 10.1016/j.ejmech.2019.111951 31821990

[B18] Orzechowska B. U. Wróbel G. Turlej E. Jatczak B. Sochocka M. Chaber R. (2020). Antitumor Effect of Baicalin from the Scutellaria Baicalensis Radix Extract in B-Acute Lymphoblastic Leukemia with Different Chromosomal Rearrangements. Int. Immunopharmacol. 79, 106114. 10.1016/j.intimp.2019.106114 31881375

[B19] Stine Z. E. Walton Z. E. Altman B. J. Hsieh A. L. Dang C. V. (2015). MYC, Metabolism, and Cancer. Cancer Discov. 5 (10), 1024–1039. 10.1158/2159-8290.CD-15-0507 26382145 PMC4592441

[B20] Tamas K. Walenkamp A. M. de Vries E. G. van Vugt M. A. Beets-Tan R. G. van Etten B. (2015). Rectal and Colon Cancer: Not Just a Different Anatomic Site. Cancer Treat. Rev. 41 (8), 671–679. 10.1016/j.ctrv.2015.06.007 26145760

[B21] Tan W. Lu J. Huang M. Li Y. Chen M. Wu G. (2011). Anti-cancer Natural Products Isolated from Chinese Medicinal Herbs. Chin. Med. 6 (1), 27. 10.1186/1749-8546-6-27 21777476 PMC3149025

[B22] Vasan N. Baselga J. Hyman D. M. (2019). A View on Drug Resistance in Cancer. Nature 575 (7782), 299–309. 10.1038/s41586-019-1730-1 31723286 PMC8008476

[B23] Verma E. Kumar A. Devi Daimary U. Parama D. Girisa S. Sethi G. (2021). Potential of Baicalein in the Prevention and Treatment of Cancer: A Scientometric Analyses Based Review. J. Funct. Foods 86, 104660. 10.1016/j.jff.2021.104660

[B24] Wang H. Li H. Chen F. Luo J. Gu J. Wang H. (2017). Baicalin Extracted from Huangqin (Radix Scutellariae Baicalensis) Induces Apoptosis in Gastric Cancer Cells by Regulating B Cell Lymphoma (Bcl-2)/Bcl-2-Associated X Protein and Activating Caspase-3 and Caspase-9. J. Tradit. Chin. Med. 37 (2), 229–235. 10.1016/s0254-6272(17)30049-3 29960296

[B25] Wang J. Wu M. Y. Tan J. Q. Li M. Lu J. H. (2019). High Content Screening for Drug Discovery from Traditional Chinese Medicine. Chin. Med. 14, 5. 10.1186/s13020-019-0228-y 30858873 PMC6394041

[B26] Wang Y. Liu Y. Du X. Ma H. Yao J. (2020). The Anti-cancer Mechanisms of Berberine: A Review. Cancer Manag. Res. 12, 695–702. 10.2147/CMAR.S242329 32099466 PMC6996556

[B27] Wu L. Wang Y. Li Z. Zhang B. Cheng Y. Fan X. (2014). Identifying Roles of "Jun-Chen-Zuo-Shi" Component Herbs of QiShenYiQi Formula in Treating Acute Myocardial Ischemia by Network Pharmacology. Chin. Med. 9, 24. 10.1186/1749-8546-9-24 25342960 PMC4196468

[B28] Xu J. Xia Z. (2019). Traditional Chinese Medicine (TCM) – Does its Contemporary Business Booming and Globalization Really Reconfirm its Medical Efficacy & Safety? Med. Drug Discov. 1, 100003. 10.1016/j.medidd.2019.100003

[B29] Xu X. H. Li T. Fong C. M. Chen X. Chen X. J. Wang Y. T. (2016). Saponins from Chinese Medicines as Anticancer Agents. Molecules 21 (10), 1326. 10.3390/molecules21101326 27782048 PMC6272920

[B30] Yeh P. Y. Chuang S. E. Yeh K. H. Song Y. C. Ea C. K. Cheng A. L. (2002). Increase of the Resistance of Human Cervical Carcinoma Cells to Cisplatin by Inhibition of the MEK to ERK Signaling Pathway Partly via Enhancement of Anticancer Drug-Induced NF Kappa B Activation. Biochem. Pharmacol. 63 (8), 1423–1430. 10.1016/s0006-2952(02)00908-5 11996883

[B31] Zhang X. Q. Yao C. Bian W. H. Chen X. Xue J. X. Zhu Z. Y. (2019). Effects of Astragaloside IV on Treatment of Breast Cancer Cells Execute Possibly through Regulation of Nrf2 via PI3K/AKT/mTOR Signaling Pathway. Food Sci. Nutr. 7 (11), 3403–3413. 10.1002/fsn3.1154 31762993 PMC6848822

[B32] Zhang Y. Liang Y. He C. (2017). Anticancer Activities and Mechanisms of Heat-Clearing and Detoxicating Traditional Chinese Herbal Medicine. Chin. Med. 12, 20. 10.1186/s13020-017-0140-2 28702078 PMC5506596

[B33] Zheng Z. Zhang W. Kong L. Liang M. Li H. Lin M. (2007). Rapid Identification of C21 Steroidal Saponins in Cynanchum Versicolor Bunge by Electrospray Ionization Multi-Stage Tandem Mass Spectrometry and Liquid Chromatography/tandem Mass Spectrometry. Rapid Commun. Mass Spectrom. 21 (3), 279–285. 10.1002/rcm.2829 17200975

[B34] Zhou W. Gross K. M. Kuperwasser C. (2019). Molecular Regulation of Snai2 in Development and Disease. J. Cell Sci. 132 (23), jcs235127. 10.1242/jcs.235127 31792043 PMC12233911

[B35] Zhou Y. Yang Z. Y. Tang R. C. (2016). Bioactive and UV Protective Silk Materials Containing Baicalin - the Multifunctional Plant Extract from Scutellaria Baicalensis Georgi. Mater Sci. Eng. C Mater Biol. Appl. 67, 336–344. 10.1016/j.msec.2016.05.063 27287129

