# Corrigendum: A novel Chinese herbal and corresponding chemical formula for cancer treatment by targeting tumor maintenance, progression, and metastasis

**DOI:** 10.3389/fphar.2023.1312683

**Published:** 2023-11-24

**Authors:** Ying-Chyi Song, Der-Yen Lee, Pei-Yen Yeh

**Affiliations:** ^1^ Graduate Institute of Integrated Medicine, College of Chinese Medicine, China Medical University, Taichung, Taiwan; ^2^ TCM Division, Jin-Mi Company, Taipei, Taiwan

**Keywords:** cancer, botanical drug, Huang-Lian (Coptis chinensis Franch.), Huang-Qin (Scutellaria baicalensis Georgi), Bai-Wei (Vincetoxicum atratum (Bunge)

In the published article, there was an error. The labeling of the pan18 cells in the source is incorrect.

A correction has been made to **Materials and Methods**, *Animal Study*, paragraph one. This sentence previously stated:

“To establish the syngeneic mouse model of pancreatic tumors, Pan18 cells (GFP-LUC-tagged) derived from pancreatic tumors in EKP (elastase-CreER; LSL-KrasG12D; p53+/−) mice, kindly gifted from Dr. Yuh-Pyng Sher (China Medical University, Taichung, Taiwan), were used.”

The corrected sentence appears below:

“To establish the syngeneic mouse model of pancreatic tumors, Pan18 cells (GFP-LUC-tagged) derived from pancreatic tumors in EKP (elastase-CreER; LSL-KrasG12D; p53+/−) mice, kindly gifted from Dr. Chia-Ning Shen (Academia Sinica, Taipei, Taiwan), were used.”

In the published article, there was another error as Dr. CN Shen was erroneously omitted from the Acknowledgments.

A correction has been made to the **Acknowledgments**. The statement previously stated:

“We thank Dr. YS Lu (Department of Oncology, National Taiwan University Hospital) for helping with the cell growth and protein expression assays.”

The corrected statement appears below:

“We thank Dr. YS Lu (Department of Oncology, National Taiwan University Hospital) for helping with the cell growth and protein expression assays. We thank Dr. CN Shen (Academia Sinica, Taipei, Taiwan) for kindly providing the Pan18 cell line.”

The authors apologize for these errors and state that this does not change the scientific conclusions of the article in any way. The original article has been updated.

