# Supplementary figures and images for: A Novel Chinese Herbal and Corresponding Chemical Formula for Cancer Treatment by Targeting Tumor Maintenance, Progression, and Metastasis
Source: Front Pharmacol. 2022 May 26;13:907826. doi: 10.3389/fphar.2022.907826 (PMC9204638; doi:10.3389/fphar.2022.907826)

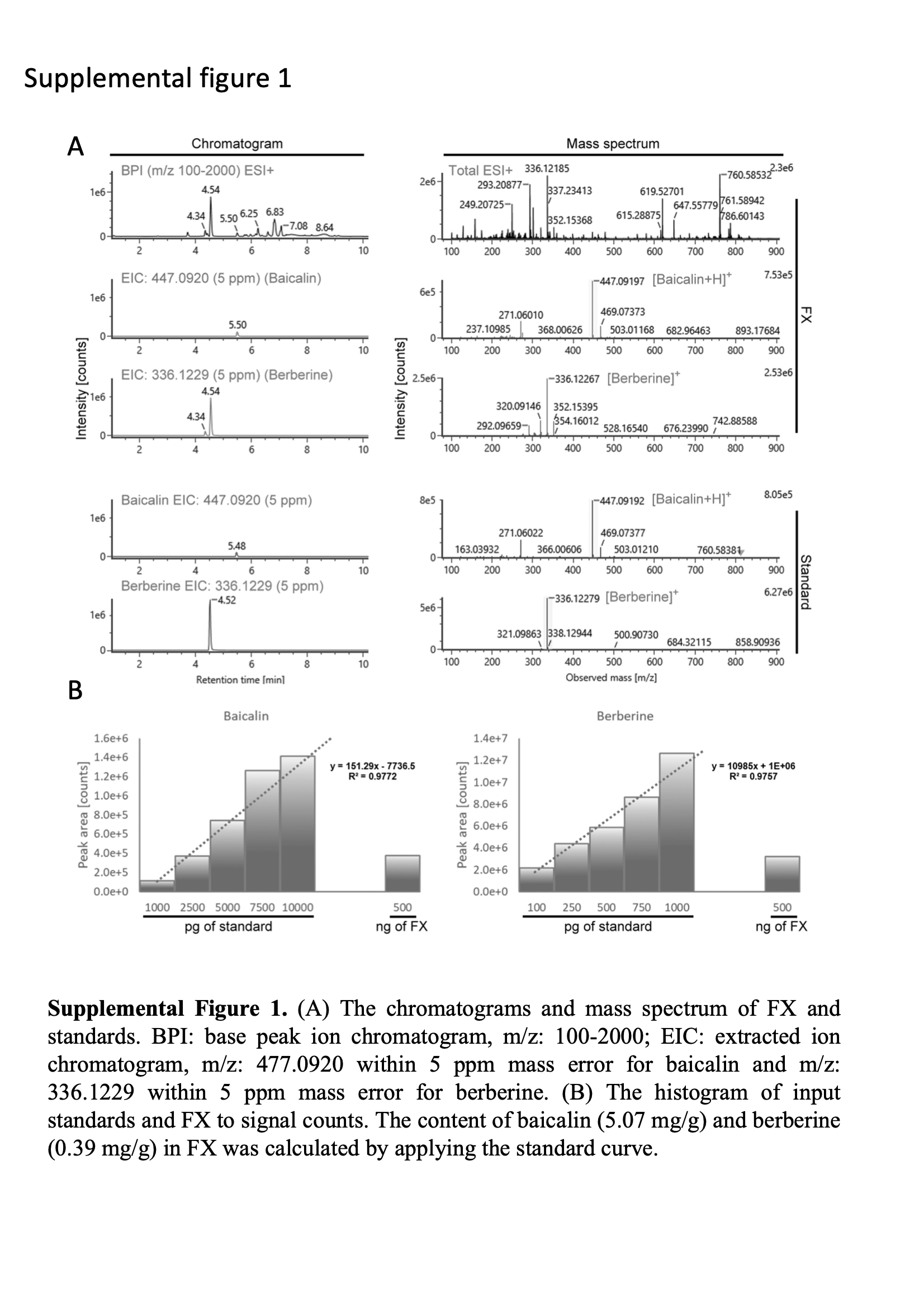

Supplement: Supplementary file 1 [file Image1.TIFF]

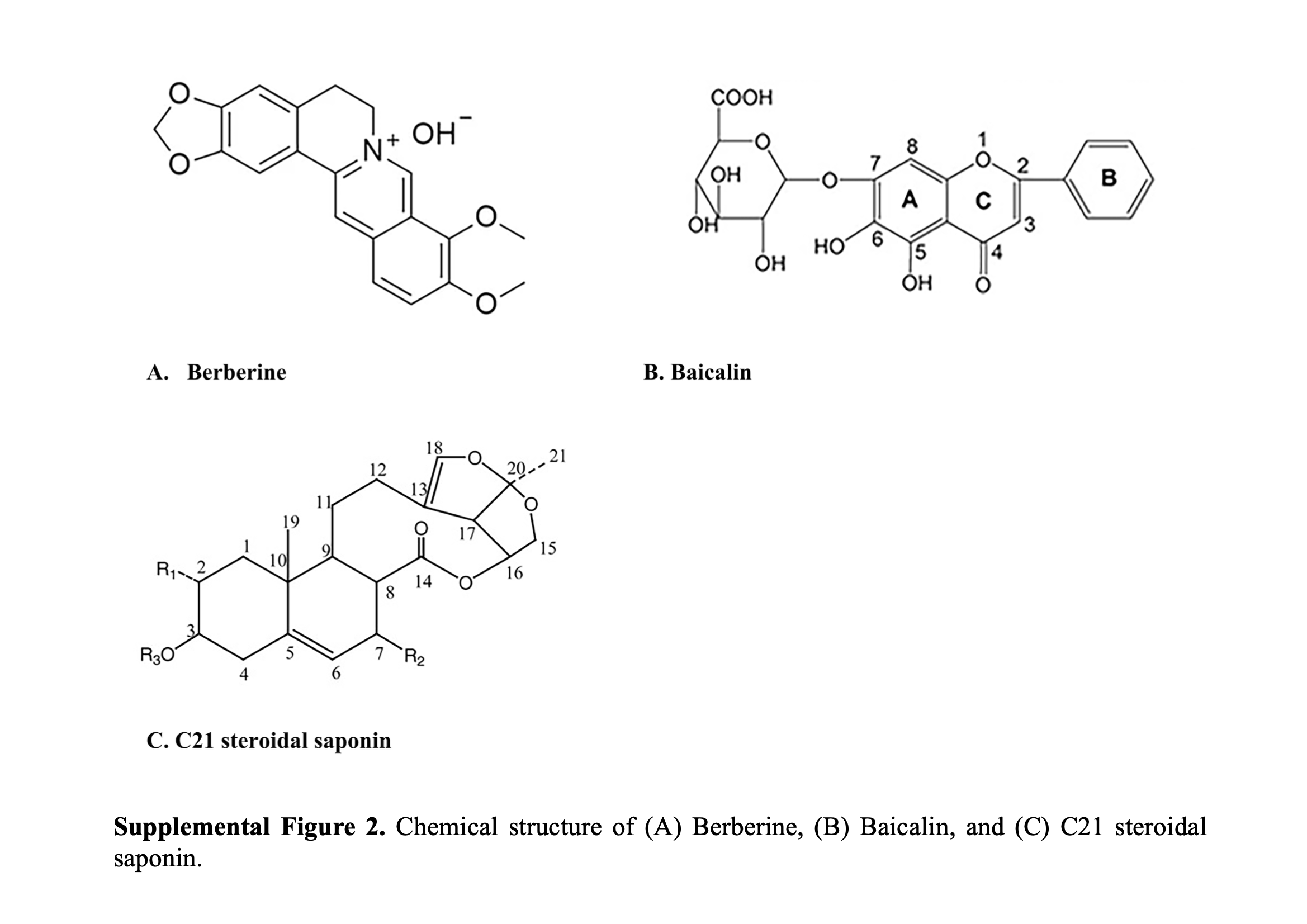

Supplement: Supplementary file 2 [file Image2.TIFF]
